# Comparison of Host Immune Responses to Homologous and Heterologous Type II Porcine Reproductive and Respiratory Syndrome Virus (PRRSV) Challenge in Vaccinated and Unvaccinated Pigs

**DOI:** 10.1155/2014/416727

**Published:** 2014-02-26

**Authors:** X. Li, A. Galliher-Beckley, L. Pappan, B. Trible, M. Kerrigan, A. Beck, R. Hesse, F. Blecha, J. C. Nietfeld, R. R. Rowland, J. Shi

**Affiliations:** ^1^Department of Anatomy and Physiology, 228 Coles Hall, College of Veterinary Medicine, Kansas State University, 1600 Denison Ave, Manhattan, KS 66506, USA; ^2^Intrexon Corporation, 108 TW Alexander Drive, Durham, NC 27709, USA; ^3^Department of Diagnostic Medicine and Pathobiology, College of Veterinary Medicine, Kansas State University, 1600 Denison Ave, Manhattan, KS 66506, USA; ^4^The Scripps Research Institute, 130 Scripps Way No. 3B3, Jupiter, FL 33487, USA

## Abstract

Porcine reproductive and respiratory syndrome (PRRS) is a high-consequence animal disease with current vaccines providing limited protection from infection due to the high degree of genetic variation of field PRRS virus. Therefore, understanding host immune responses elicited by different PRRSV strains will facilitate the development of more effective vaccines. Using IngelVac modified live PRRSV vaccine (MLV), its parental strain VR-2332, and the heterologous KS-06-72109 strain (a Kansas isolate of PRRSV), we compared immune responses induced by vaccination and/or PRRSV infection. Our results showed that MLV can provide complete protection from homologous virus (VR-2332) and partial protection from heterologous (KS-06) challenge. The protection was associated with the levels of PRRSV neutralizing antibodies at the time of challenge, with vaccinated pigs having higher titers to VR-2332 compared to KS-06 strain. Challenge strain did not alter the cytokine expression profiles in the serum of vaccinated pigs or subpopulations of T cells. However, higher frequencies of IFN-**γ**-secreting PBMCs were generated from pigs challenged with heterologous PRRSV in a recall response when PBMCs were re-stimulated with PRRSV. Thus, this study indicates that serum neutralizing antibody titers are associated with PRRSV vaccination-induced protection against homologous and heterologous challenge.

## 1. Introduction

Porcine reproductive and respiratory syndrome (PRRS) is an economically important pandemic disease characterized by reproductive failure in sows and respiratory disease in young pigs. A recent study estimates that the total productivity losses in the U.S. swine industry due to PRRS are currently $664 million annually, an increase from the $560 million annual cost estimated in 2005 [1]. This indicates that not only does PRRS have a significant financial impact on the pork industry but also current strategies for reducing the burden of PRRS virus are not adequate.

PRRS is caused by porcine reproductive and respiratory syndrome virus (PRRSV), which is a member of the genus *Arterivirus*, family *Arteriviridae,* and order *Nidovirales*. PRRSV is known to mutate rapidly in both *in vitro* cell culture models and *in vivo* in natural field infections [2]. The ability of PRRSV to mutate rapidly creates genetically extensive and antigenic diverse strains in both North American and European field isolates [3]. The high genetic mutation rate of PRRSV poses a challenge for PRRSV vaccine development [2]. Currently, both inactivated PRRSV vaccines and modified live virus (MLV) PRRSV vaccines are widely used to control the disease. However, inactivated vaccines as well as modified live vaccines have been shown to be ineffective in providing protective immunity to heterologous strains of PRRSV at the herd level [4–7]. Therefore, development of a broadly protective PRRSV vaccine will be one of the most efficient solutions to control the prevalence of PRRS worldwide.

It has been shown that pigs infected with PRRSV have inadequate immune responses, such as delayed onset of neutralizing antibody as well as weak interferon (IFN)-*γ* responses [2, 8]. Development of different types of vaccines aiming to increase host immune response and get broader protection from various field PRRSV infections has been proposed [9]. Currently, PRRSV-MLV is used to control the disease worldwide. However, the high incidence of genetic mutation during PRRSV transmission often results in vaccines based on strains of PPRSV isolated twenty years ago, such as MLV, having limited protection from new emerging viral strains. Disparity of immune responses elicited by different PRRSV strains was reported previously [10]. However, the role of humoral and cellular immune responses was not clearly elucidated in these reports with regard to the protection from virus challenge with different PRRSV strains. Therefore, dissecting the mechanisms of immune responses that are predictive of protection against heterologous PRRSV challenge will be valuable for the development of more efficacious vaccines. In this study, we investigated the differential profiles of host immune responses in naive or vaccinated pigs challenged with homologous and heterologous PRRSV strains.

## 2. Materials and Methods

### 2.1. Cells and Virus

MARC-145 cells were maintained in Modified Eagle's medium (MEM) supplemented with 7% fetal bovine serum (FBS) containing 100 U penicillin/mL and 100 *μ*g streptomycin/mL at 37°C with 5% CO_2_. Virus stocks were prepared and titered in MARC-145 cells and stored in aliquots at −80°C until use. For virus infection and titration, MEM supplemented with 2% FBS was used. PRRS modified live virus vaccine (Ingelvac PRRS MLV) was purchased from Boehringer Ingelheim Vetmedica Inc. PRRSV strains VR-2332, KS-06-72109 (KS-06), and NVSL97-7895 have been described previously [11, 12].

### 2.2. Pigs, Vaccination, and Challenge

**Figure 1:**
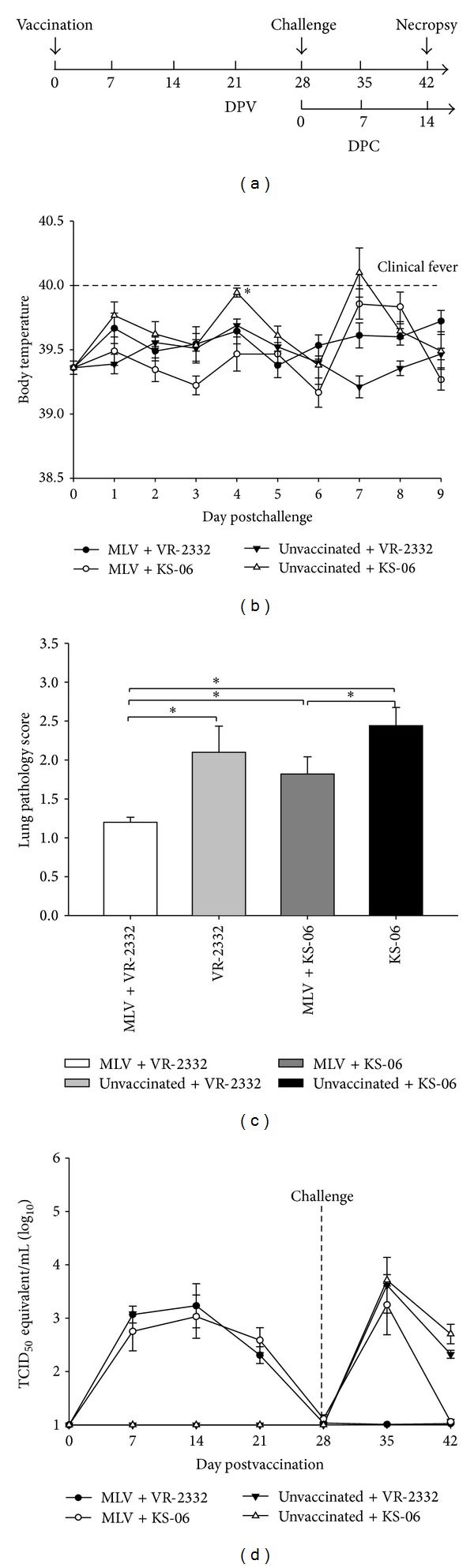
Vaccination with PRRSV-MLV induced complete protection from homologous PRRSV challenge and partial protection from heterologous challenge. (a) Experimental timeline. (b) Rectal temperature of pigs was monitored daily after PRRSV challenge. (c) Gross lung lesion scores present in all lung lobes on DPC 14 were scored using a 4-point scale. (d) PRRSV viral RNA in the serum throughout the study was determined by qPCR. Each bar represents the average of samples from five pigs ± SEM. *P < 0.05.

Twenty conventional large White-Duroc crossbred weaned specific pathogen-free piglets (3 weeks of age) were divided into four groups within the Large Animal Research Center (LARC) facility, Kansas State University. These piglets were confirmed sera-negative for antibodies to PRRSV by ELISA and PRPSV-free in the blood by RT-PCR. Pigs were allowed to acclimate for an additional week before initiation of the experiment. The first two groups were immunized intramuscularly on day postvaccination (DPV) 0 with vaccine (PRRS-MLV, 1 × 10^6^ TCID_50_/pig). The other two groups were used as control groups before challenge and remained unvaccinated (Figure 1(a)). After four weeks the pigs were challenged with 2 × 10^5^ TCID_50_/pig of VR-2332 or KS-06 PRRSV. Necropsy was performed at 14 days postchallenge (DPC). Pigs were monitored for rectal temperature for the first 9 days after challenge and body weight once a week for the duration of this experiment.

### 2.3. Collection of Blood Samples for Analysis

Blood was collected on DPV 0, 7, 14, 21, and 28 and DPC 7 and 14. Serum was separated from clotted blood and preserved at −20°C. Serum was used for evaluation of viral titers, serum neutralizing antibody titers, PRRSV-specific ELISA antibody titers (Herdchek Porcine Reproductive and Respiratory Syndrome Antibody test Kit, IDEXX Laboratories), and cytokine expression as described previously [12]. Peripheral blood mononuclear cells (PBMCs) were isolated from heparinized blood samples by Ficoll-Hypaque gradient centrifugation using Histopaque-1077 (Sigma-Aldrich, St. Louis, MO). PBMCs were used for ELISpot assay and flow cytometry analysis as described previously [12].

### 2.4. Gross Lung Lesion Analysis

Pigs were humanely euthanized on DPC 14 as approved by the Kansas state University Institutional Animal Use and Biosafety Committee. The lungs were macroscopically and microscopically evaluated as previously described [13]. Briefly, the dorsal and ventral surfaces of each lung lobe were given a score representing the approximate proportion that was consolidated. Individual lobe scores were used to determine an overall lung score representing the percentage of interstitial pneumonia. Sections of each of the 4 lobes of the right lung were fixed in 10% buffered neutral formalin, paraffin-embedded, sectioned, and stained with hematoxylin and eosin (H & E). Scoring of microscopic lung pathology was done in a blinded fashion by two veterinary pathologists in the Kansas State Veterinary Diagnostic Laboratory. Grading was on a 4 point scale as previously described [13].

### 2.5. Analysis of PRRSV Circulating in the Blood

Total RNA was extracted from pig serum and one-step SyBR Green real-time PCR (Bio-Rad) was performed to evaluate the PRRSV ORF7 expression level as previously described [14]. For quantification, total RNA of a known TCID_50_ of virus was 10-fold serially diluted and was used to generate a standard curve. The virus quantities of unknown samples were determined by linear extrapolation of the Ct value plotted against the standard curve.

### 2.6. Virus Neutralizing Antibody Titer

Virus neutralizing antibody titers were assayed as previously described [12, 14]. Briefly, serum samples were heat inactivated (56°C, 30 min) and serially diluted before the titration. The serial dilutions of serum samples were mixed with equal volume of PRRSV strains: VR-2332, KS-06, or NVSL97-7895 containing 200 TCID_50_ of the virus. After incubation at 37°C for 1 h, the mixtures were transferred to MARC-145 monolayers in 96-well plates and incubated for an additional 72 h at 37°C in a humidified atmosphere containing 5% CO_2_. Cells were then examined for cytopathic effects (CPE). CPE was used to determine the end-point titers that were calculated as the reciprocal of the highest serum dilution required to neutralize 200 TCID_50_ of PRRSV in 90% of the wells.

### 2.7. ELISpot Assay

Half million PBMCs were plated in enriched RPMI in a 96-well multiscreen plate (Millipore, Billerica, MA) precoated overnight with capture IFN-*γ* mAB (BD Pharmingen, San Diego, CA). PBMCs were restimulated with three different strains of PRRSV (VR-2332, KS-06, or NVSL97-7895) at 0.1 MOI for 24 h at 37°C. IFN-*γ*-secreting cells were detected by biotinylated anti-pig IFN-*γ* detection antibody and visualized using the immunospot image analyzer (Cellular Technology, Cleveland, OH). We calculated the number of PRRSV-specific IFN-*γ*-secreting cells by subtracting the number of spots in unstimulated cultures (all samples were <10) from the count of PRRSV-stimulated cultures. Data were presented as the mean numbers of antigen-specific IFN-*γ*-secreting cells per 10^6^ PBMCs from duplicate wells of each sample.

### 2.8. Flow Cytometry Analysis

Flow cytometry analysis was performed to determine different lymphocyte populations based on the cell surface marker phenotype: T-helper cells (CD3^+^CD4^+^CD8^−^), cytotoxic T lymphocytes (CD3^+^CD4^−^CD8^+^), Th/memory cells (CD3^+^CD4^+^CD8^+^), and *γδ* T cells (CD8^+^TcR1N4^+^). Mouse anti-pig TcR1N4 antibody was purchased from VMRD (Pullman, WA), and the rest of the antibodies used in this study were purchased from BD Biosciences. Immunostained cells were acquired using a FACS Caliber (BD Biosciences) flow cytometer as described previously [12, 14]. Briefly, PBMC was treated with 2% pig serum to block Fc receptors. Cells were then stained with an appropriate Ab which was either directly conjugated to a specific fluorochrome or with a purified Ab to pig specific immune cell surface marker (TcR1N4). For cells stained with a purified Ab, labeled cells were treated with antispecies isotype specific secondary Ab conjugated with fluorochrome. Finally, cells were fixed with 1% paraformaldehyde before reading on a flow cytometer. Percentages of each lymphocyte population were analyzed by 100,000 unique events using FlowJo software (Tree Star, Inc., OR, USA).

### 2.9. Analysis of Cytokine Responses

Pig sera were collected at DPC 7 to evaluate IL-4, IL-8, IL-10, IFN-*γ*, TNF-*α* (Life Technologies, Carlsbad, CA), and IFN-*α* (Abcam, Cambridge, MA) secretion profiles by ELISA. Procedures were performed as per the manufacturer's instructions. For a given sample, the OD_450_ was then transformed to concentration by applying a linear regression formula calculated from the results of the standards provided in each kit.

### 2.10. Statistical Analysis

All data were expressed as the mean value of five pigs ± SEM. The differences in the level of body temperature, lung pathology score, humoral response, cytokine production, and viremia among each group were determined by the one-way analysis of variance (ANOVA) followed by post-hoc Tukey's test using SigmaPlot 11 software (Systat Software Inc., San Jose, CA). The difference in the percentage of different T cell subpopulations was determined by the paired *t*-test using SigmaPlot 11 software.

## 3. Results

### 3.1. Vaccination with PRRSV-MLV Induced Complete Protection from Homologous PRRSV Challenge and Partial Protection from Heterologous Challenge

To compare host immune responses to challenge by different PRRSV isolates, pigs were either vaccinated with PRRSV-MLV or a mock vaccine (PBS) on day 0 and then challenged with homologous VR-2332 or heterologous KS-06 PRRSV on day 28 (Figure 1(a)). Clinically, the mean body temperature of unvaccinated pigs challenged with the KS-06 strain of PRRSV was higher compared to that in the other three groups at DPC 4 (Figure 1(b)). The body weight of all pigs was tracked throughout the study and weights of all groups were similar during the vaccination phase. Interestingly, pigs vaccinated with MLV and challenged with VR-2332 had a slightly higher weight gain than that of the other groups on DPC 14 (data not shown). Unvaccinated pigs that were challenged with either VR-2332 or the KS-06 strain had higher lung lesion scores on DPC 14 compared to those in vaccinated pigs (Figure 1(c)). Vaccinated pigs challenged with VR-2332 showed full protection against PRRSV with average lung scores being normal and no lung damage observed during pathological analysis. Additionally, vaccinated pigs challenged with the KS-06 strain had moderate protection as shown by decreased lung scores compared to that in unvaccinated-KS-06 challenged pigs (Figure 1(c)).

In addition, complete protection in vaccinated pigs against homologous challenge was confirmed with the absence of PRRS viral RNA in the serum on DPC 7. As shown in Figure 1(d), pigs vaccinated with MLV had efficiently cleared the VR-2332 challenge virus from the blood to undetectable levels. Vaccinated pigs challenged with the KS-06 strain had less circulating PRRSV in the blood than that in unvaccinated-KS-06 challenged pigs, but the difference was not statistically significant. By DPC 14, the levels of PRRSV virus circulating in the blood were reduced significantly in all vaccinated groups. Therefore, our results suggest that PRRSV-MLV can protect pigs from homologous challenge and provide moderate protection against heterologous PRRSV challenge.

### 3.2. Serum Neutralizing Antibody Titer Is Associated with PRRSV Vaccination-Induced Protection against Homologous and Heterologous Challenge

**Figure 2:**
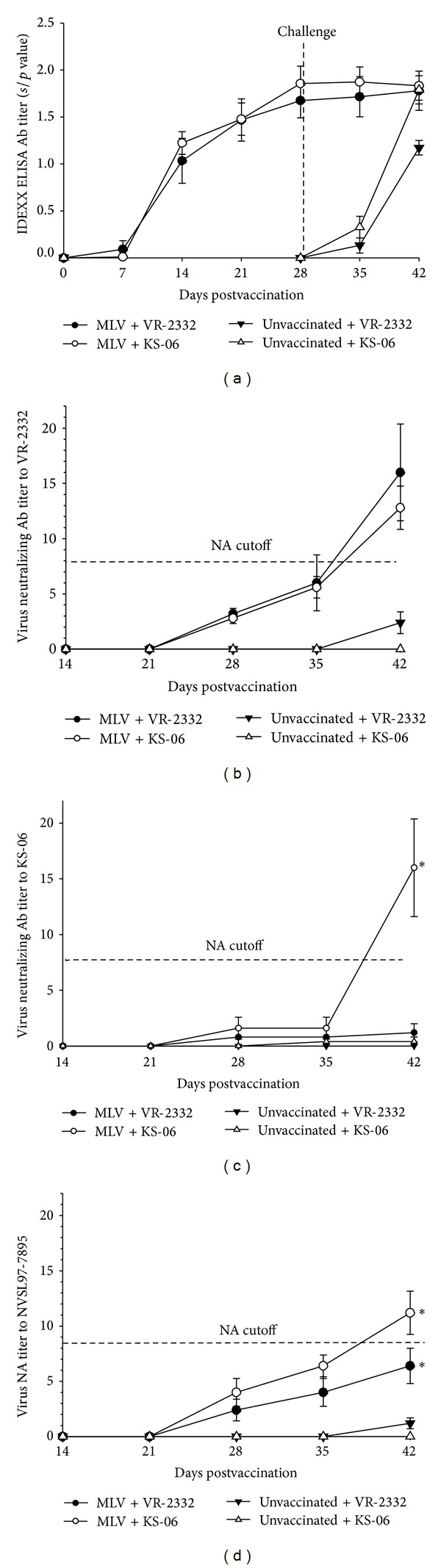
Serum neutralizing antibody titer is associated with PRRSV vaccination-induced protection against homologous and heterologous challenge. (a) PRRSV-specific antibodies were detected in the serum using IDEXX ELISA kit. The threshold for positive sera was set at a sample to positive (*s*/*p*) ratio of 0.4 according to the manufacturer's instructions. ((b)–(d)) Serum samples were titrated on MARC-145 cells and the levels of anti-PRRSV neutralizing Abs were determined as the reciprocal of the highest dilution that could inhibit CPE. Data were shown as mean ± SEM for 5 pigs per group. *P < 0.05.

It has been shown that a vigorous anti-PRRSV antibody response in pigs is seen early after vaccination or PRRSV exposure [15]. To determine antibody responses, we analyzed PRRSV-specific ELISA antibodies in homologous- and heterologous-challenged pigs using commercial IDEXX ELISA kit. Serum samples were collected at various time points and used to determine the PRRSV-specific antibody levels. As shown in Figure 2(a), vaccinated pigs produced PRRSV-specific antibodies starting from DPV 14. Interestingly, the antibody titers in vaccinated pigs were not further enhanced by PRRSV challenge. Additionally, it was found that unvaccinated pigs challenged with the KS-06 isolate showed a faster onset and higher ELISA antibody titers than unvaccinated pigs challenged with VR-2332 (Figure 2(a)).

However, there is no evidence that early anti-PRRSV antibody response plays a role in the protection against PRRSV infection. In contrast, later appearing antibodies with PRRSV neutralizing activity have been shown to play a critical role in anti-PRRS immunity. A previous study showed that passive transfer of neutralizing antibodies with a titer of 8 to recipient piglets protected them from challenge-induced viremia, while transfer of serum titers of 32 produced sterilizing immunity [15], suggesting that neutralizing antibody titers over 8 can protect pigs from PRRSV. The ability of a vaccine (modified live or inactivated) to induce PRRSV neutralizing antibodies to specific PRRSV isolates influences the level of protection the vaccinated pig has to the specific challenge strain [15, 16]. Therefore, we analyzed the PRRS virus neutralizing antibody (VN) titers in the serum of different treatment groups. As shown in Figure 2(b), MLV vaccinated pigs began to develop VN titers to VR-2332 at DPV 28 and the titers were significantly higher at the end of the study as compared to those in unvaccinated pigs. It is worth noting that high titer of VN antibodies against the KS-06 stain was detected only in pigs vaccinated with MLV but not in unvaccinated pigs after both groups of pigs were challenged with the KS-06 strain (Figure 2(c)). To assay for broad neutralizing activity, another PRRSV strain, NVSL97-7895, was used to measure the VN titer of all serum samples. As shown in Figure 2(d), VN antibodies against NVSL97-7895 were developed only in vaccinated pigs, and the serum VN titers in vaccinated pigs challenged with the KS-06 strain were higher than those in vaccinated pigs challenged with the homologous VR-2332. This indicates that pigs receiving vaccination followed by challenge with a different strain of PRRSV may generate antibodies with a broader neutralizing spectrum.

### 3.3. PRRSV-Dependent Cytokine Expression Patterns Are PRRSV Challenge Strain Specific

**Figure 3:**
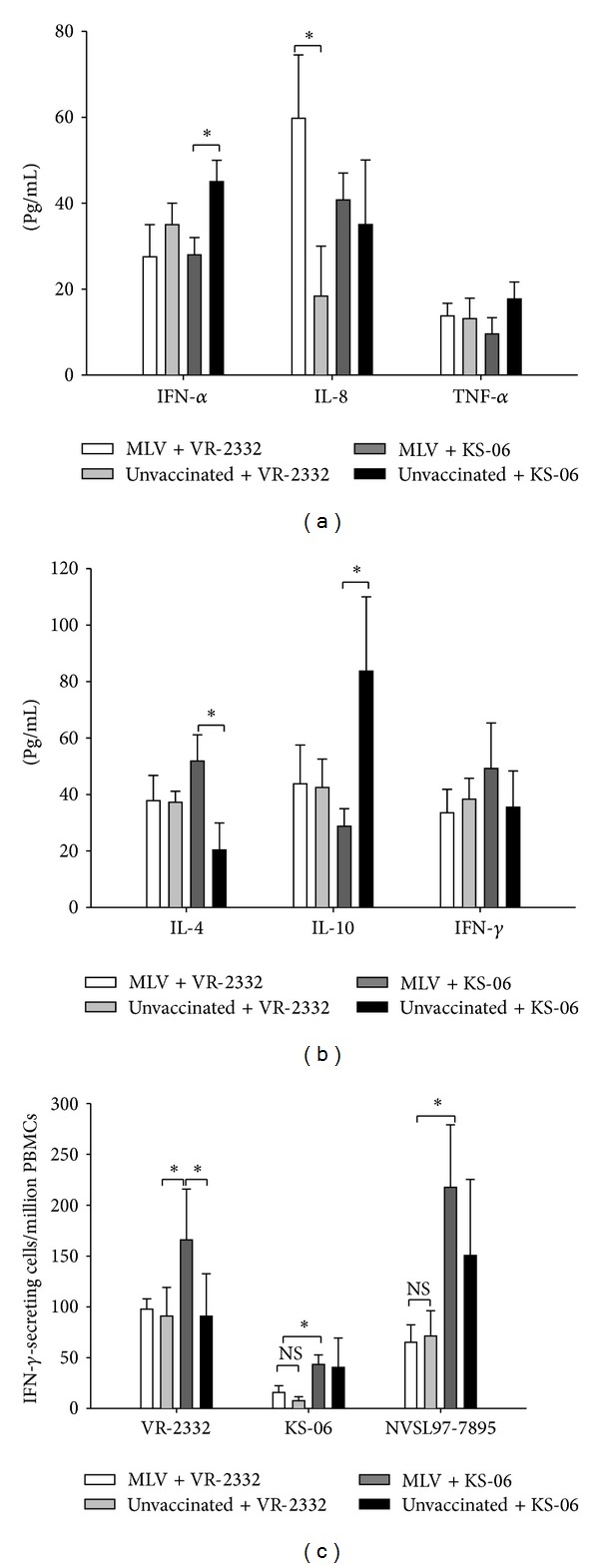
PRRSV-dependent cytokine expression patterns are PRRSV challenge strain specific. (a) Innate and (b) adaptive cytokine expression profiles in the sera of pigs at DPC 7 were tested by ELISA. (c) PBMCs collected at DPC 14 were restimulated with VR-2332, KS-06, or NVSL97-7895 strains of PRRSV. IFN-*γ*-secreting cells were then analyzed by ELISpot assay. Data were shown as mean ± SEM for 5 pigs per group. *P < 0.05. NS: not significant.

Compared to MLV vaccinated pigs challenged with the KS-06 strain, unvaccinated pigs displayed significantly higher IFN-*α* level in the serum (Figure 3(a)). In contrast, the difference in IFN-*α* production was not detected between vaccinated and unvaccinated pigs after they were challenged with VR-2332. Interestingly, vaccinated pigs produced significant higher levels of IL-8 compared to unvaccinated pigs after they were challenged with VR-2332 (Figure 3(a)). TNF-*α* expression levels were low in all pigs and there was no significant difference among treatment groups. Furthermore, serum IL-10 levels were significantly higher in unvaccinated pigs after KS-06 PRRSV challenge than those in vaccinated pigs (Figure 3(b)). In contrast, vaccinated pigs displayed a higher level of serum IL-4 after VR-2332 challenge compared to unvaccinated pigs (Figure 3(b)). There was no significant difference in serum levels of IFN-*γ* among all treatment groups.

Vaccination with PRRS-MLV has been shown to induce the production of IFN-*γ*-secreting cells as a mechanism of protecting pigs against PRRSV viremia [17]. Therefore, the frequency of IFN-*γ*-secreting cells in PBMCs was evaluated on DPC 14 in a recall response in which PBMCs were restimulated with VR-2332, KS-06, or NVSL97-7895 PRRSV. As shown in Figure 3(c), when restimulated with VR-2332, PBMCs from vaccinated pigs challenged with the KS-06 strain developed more IFN-*γ*-secreting cells than those from the other three groups. When restimulated with KS-06 or NVSL97-7895, PBMCs from KS-06 challenged pigs produced significantly higher amount of IFN-*γ*-secreting cells than that from pigs challenged with VR-2332. Finally, the ratios of IFN-*γ*-secreting cells in PBMCs restimulated with KS-06 PRRSV in all treatment groups were significantly lower than those in PBMCs restimulated with VR-2332 or NVSL97-7895.

### 3.4. T Lymphocyte Subpopulations Vary between Unvaccinated and Vaccinated Groups and Are Independent of PRRSV Challenge Strain

**Figure 4:**
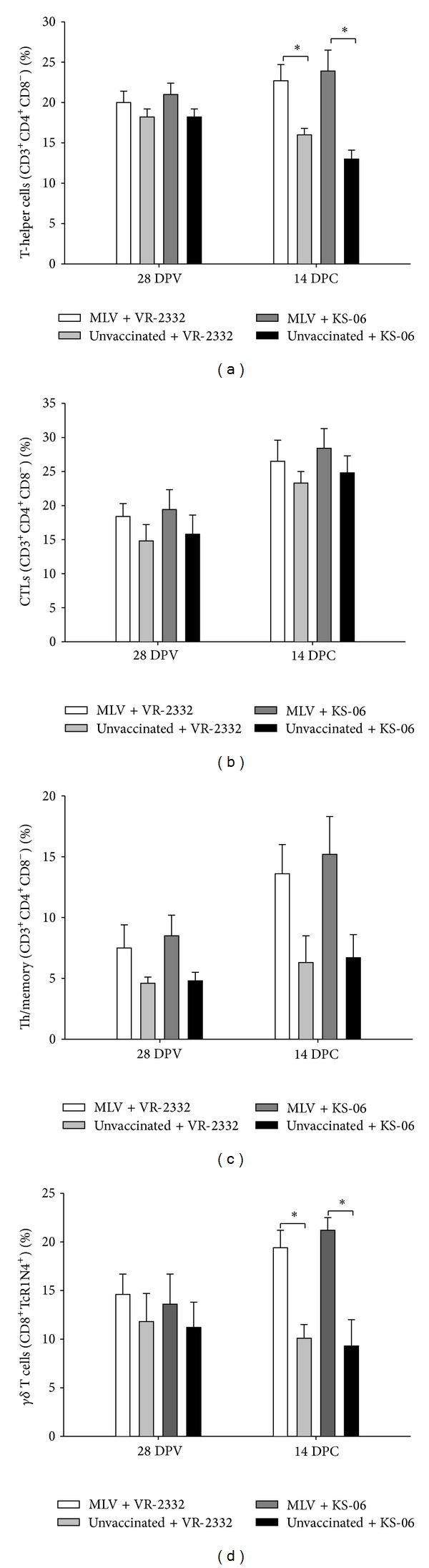
T lymphocyte subpopulations vary between unvaccinated and vaccinated groups and are independent of PRRSV challenge strain. PBMCs were isolated from pigs at necropsy (DPC 14) and T cell subsets were determined by flow cytometry analysis according to their phenotypes. Shown are the percentages of (a) T-helper cells that were CD3^+^/CD4^+^/CD8^−^, (b) Cytotoxic T lymphocytes that were CD^+^CD4^−^CD8^+^, (c) Th/memory cells that were CD3^+^CD4^+^CD8^+^, and (d) *γδ* T cells that were CD8^+^TcR1N4^+^. Data were shown as mean ± SEM for 5 pigs per group. *P < 0.05.

T lymphocyte subpopulations are reported to vary in pigs after challenge with different PRRSV strains [18]. In this study, we evaluated the changes in frequency of various lymphocyte populations before and after PRRSV challenge in all experimental groups. On DPV 28, the frequencies of T-helper cells (Figure 4(a)), cytotoxic T lymphocytes (CTLs; Figure 4(b)), and *γδ* T cells (Figure 4(d)) in PBMCs were similar in vaccinated and unvaccinated pigs, while the frequencies of Th/memory cells in unvaccinated pigs were lower compared to those in vaccinated pigs (Figure 4(c)). On DPC 14, the frequencies of T-helper, Th/memory, and *γδ* T cells in PBMCs from vaccinated pigs were higher than those from unvaccinated pigs. It is worth noting that the frequencies of various T cell populations in PBMCs from vaccinated or unvaccinated pigs were not affected by the difference in challenge strains (VR-2332 versus KS-06), suggesting that PRRSV challenge strain does not affect T cell subpopulations.

## 4. Discussion

As one of the most prevalent diseases in swine, PRRS has caused vast economic losses to the pig industry worldwide. Adding to its devastation, the rapid evolution rate of PRRS virus generates countless genetically distinct field isolates, many of which have increased pathogenic ability [2, 10, 18]. Recent outbreaks of PRRSV in China were characterized by high morbidity/mortality and commercially available PRRSV vaccines offered no protection [19, 20]. This demonstrates that current commercial vaccines offer limited or no protection from newly emerging PPRSV field strains. Therefore, studies on the difference of immune responses to homologous and heterologous challenge lay an important foundation for the development of effective vaccines and eradiation strategies. The present study evaluated the differences of immune responses between vaccinated and unvaccinated pigs when challenged with homologous or heterologous PRRSV. Here we demonstrate that serum neutralizing antibody titers are associated with PRRSV vaccination-induced protection against homologous and heterologous challenge.

A recent review suggests that antibodies directed against both nonstructural and structural proteins including NSP2, GP2, GP4, and GP5 may possess PRRSV neutralizing activity [5], and the variability within GP5 may explain the deficiency in cross-protection of current vaccines against heterologous strains of PRRSV. VR-2332 (homologous) and KS-06 strain (heterologous), the PRRS viruses used for challenge experiments in this study, share 99.7% or 90.2% similarity with the PRRSV-MLV vaccine strain based on GP5 amino acid sequence, respectively. From gross lung pathology and viremia results, homologous VR-2332 PRRSV infection was fully prevented after vaccination with PRRSV-MLV as evidenced by lack of virus in sera on DPC 7 and normal gross lung pathology scores (Figures 1(c) and 1(d)). Gross lung pathology scores in the vaccinated pigs challenged with the KS-06 strain were decreased compared to those in the unvaccinated pigs, which indicate MLV vaccination can lead to partial protection from heterologous PRRSV. These results allow us to compare the immune responses from pigs with complete, partial, and no (unvaccinated) protection against PRRSV challenge.

By DPV 14, antibodies specific for N proteins of PRRSV, as measured by the IDEXX ELISA kit, were detected in vaccinated pigs and increased throughout the experimental period. PRRSV-specific antibodies were similar between vaccinated groups throughout the study, suggesting that anti-N protein antibodies are not predictive of PRRSV protection. Interestingly, we did observe that KS-06 PRRSV challenge induced a faster anti-PRRSV antibody response as compared to the vaccine strain, suggesting that more virulent strains could induce a stronger antibody response.

In contrast to anti-N protein antibodies, virus neutralizing antibodies (VNs) have been shown to correlate with protection from PRRSV [15, 16, 21]. We found that VNs to different PRRSV strains did not start to emerge until DPV 28 in the vaccinated pigs. At the time of PRRSV challenge (DPV 28), vaccinated pigs developed higher VN titers to VR-2332 (Figure 2(b)) than to KS-06 strain (Figure 2(c)), suggesting an association between PRRSV strain-specific VN titer and level of protection from PRRSV. Vaccinated pigs did not develop VNs to KS-06 after vaccination but developed significantly higher VN titers to KS-06 as compared to the other three groups two weeks after challenge, which suggests that the KS-06 specific VN could be induced by KS-06 challenge (Figure 2(c)). Also, vaccinated and KS-06 challenged pigs developed a higher level of VN antibodies to the heterologous NVSL97-7895 PRRSV strain (Figure 2(d)). This result supports the notion that two vaccinations with different PRRSV strains can generate higher neutralizing Abs and broader cross-protection against various PRRSV field strains. Similar observation has been reported in influenza virus vaccination strategy studies [22].

It was reported that PRRSV can inhibit the expression of IFN-*α* [23]. However, we found that the level of IFN-*α* was increased in unvaccinated pigs challenged with KS-06 virus. Similar to previous reports, serum level of IFN-*α* is not associated with the PRRS virus clearance in pigs after viral challenges [18]. The serum level of inflammatory cytokine IL-8 in vaccinated pigs challenged with homologous VR-2332 virus was the highest among all treatment groups (Figure 3(a)). Our results are consistent with previous studies which have shown that low level of serum IL-8 is seen in persistent PRRSV infection, and elevated IL-8 levels in serum are correlated with the clearance of PRRS virus [24]. However, it remains to be determined how elevated IL-8 may contribute to the clearance of PRRS virus in vaccinated pigs and whether the level of serum IL-8 can be used to predict vaccination-induced protection in pigs.

The expression of IL-4 was significantly higher in vaccinated pigs as compared to that in unvaccinated pigs after KS-06 challenge. This and our previous study [12] and results from others [25] suggest that increased IL-4 expression may play a positive role in vaccination-mediated clearance of heterologous PRRS virus. However, IL-4 level in the serum may not have a direct role in protecting pigs from PRRSV infection since pigs challenged with homologous PRRSV (VR-2332) did not show increased IL-4 production. Thus, whether or not IL-4 plays an important role in the development of vaccination-induced protection against PRRSV has yet to be explored in future studies.

PRRSV infection has been shown to induce a strong immunosuppressive response characterized by promoting the secretion of IL-10 to antagonize the protective Th1 immune response [26]. In our study, we found that IL-10 production in the serum was increased in unvaccinated pigs, but not in vaccinated pigs, when they were challenged with the KS-06 strain (Figure 3(b)). In contrast, both unvaccinated and vaccinated pigs challenged with VR-2332 had similar levels of serum IL-10. The level of serum IL-10 in PRRS infection has been reported to be virus strain-dependent, which may be related to the virulence of each viral isolate [26]. Thus, the difference in IL-10 production between the two challenged groups may be due to the fact that the KS-06 isolate is more virulent than the VR-2332 isolate.

IFN-*γ* is a key cytokine that is associated with host cell-mediated immunity (CMI) response, which is secreted by natural killer cells and several different T cell subpopulations. A report by Xiao et al. shows that the level of IFN-*γ* expression after PRRSV infection was variable and did not correlate with virus load [27]. Similar to their findings, we did not observe any changes to serum levels of IFN-*γ* among the four treatment groups (Figure 3(b)). In a recall response, IFN-*γ*-secreting cells from memory lymphocytes were calculated by stimulating PBMCs with different PRRSV isolates. MLV vaccination generated higher frequency of IFN-*γ*-secreting cells. However, PBMCs isolated from vaccinated and KS-06 challenged pigs generated more IFN-*γ*-secreting cells when restimulated with homologous or heterologous PRRSV as compared to those from unvaccinated pigs (Figure 3(c)). We found that the lowest number of IFN-*γ*-secreting cells was from PBMCs restimulated with the KS-06 strain, as compared to another heterologous strain NVSL97-7895 or VR-2332 stimulation. This may be due to the fact that the KS-06 isolate is more virulent than the other two strains and can cause a stronger immunosuppression during infection [18]. Our results suggest that increased IFN-*γ* expression does not correlate with protection against PRRSV as evidenced by lower levels of IFN-*γ* in fully protected vaccinated pigs challenged with VR-2332 compared to partially protected vaccinated pigs challenged with KS-06 strain. Therefore, the role of IFN-*γ* in the protection from PRRSV infection needs to be further explored.

A high frequency of *γδ* T cells in pigs is considered to be related to the activation status of the innate immune system, and CD4^+^CD8^+^ double positive T cells possess memory, T-helper, and cytolytic properties [28, 29]. Although significant increases in the frequency of T-helper, Th/memory, and *γδ* T cells in PBMCs were observed in vaccinated pigs compared to that in unvaccinated pigs, and this may suggest a protective role of these cells against PRRSV infection, this parameter cannot predict the level of protection since changes in T cell subpopulations are similar between fully and partially protected groups of pigs.

## 5. Conclusion

Difference of immune responses between vaccinated and unvaccinated pigs challenged with either homologous or heterologous PRRSV has been presented in this study. A better understanding of immune response profiles leading to full and partial protection from PRRSV challenge will facilitate the development of more efficacious vaccines for a broader cross-protection as well as new strategies combating various circulating PRRS virus strains.
